# Migratory birds as a potential overseas transmitter of Shiga toxin-producing *Escherichia coli*

**DOI:** 10.1080/23144599.2021.1989937

**Published:** 2021-10-26

**Authors:** Aya Seleem, Maha A. Sabry, Khaled A. Abdel-Moein

**Affiliations:** Department of Zoonoses, Faculty of Veterinary Medicine, Cairo University, Cairo, Egypt

**Keywords:** Shiga toxin-producing*E. coli*, migratory birds, public health

## Abstract

Shiga toxin-producing *Escherichia coli* (STEC) has a great public health importance. This study was conducted to investigate the potential role of migratory birds in the transmission of STEC. For this purpose, cloacal swabs were collected from 349 migratory birds (209 ducks and 140 quails) from Damietta governorate, Egypt. The collected swabs were cultured for isolation of STEC using the STEC CHROMagar. STEC isolates were identified based on colonial characteristics, Gram’s stain, conventional biochemical tests and molecular detection of *stx1, stx2* and *eae* genes. Positive isolates were serotyped and examined for their antibiotic susceptibility pattern. Furthermore, gene sequencing was performed for genes *stx1*and *stx2*. Of the examined birds, two STEC isolates were a obtained with an overall occurrence rate 0.57% (2/349), one isolate carried *stx2* gene from a migratory quail 0.71% (1/140), and another isolate from a migratory duck carried *stx1* gene 0.48% (1/209), whereas both isolates were negative for *eae* gene. Moreover, the duck isolate was serotyped O86, while the quail isolate was serotyped O125; both isolates were multidrug resistant. The phylogenetic analysis of the obtained *stx1* and *stx2* genes revealed high genetic relatedness to those isolated from human cases in the countries where such birds either lived or were in their migratory pathway. In conclusion, this study highlights the potential role of migratory birds in transmitting multidrug-resistant STEC across their migratory pathway.

## 1. Introduction

Bird migration from their natural habitat to another niche during certain seasons is a natural phenomenon that is repeated every year. Lack of food and breeding habitat promote the birds to migrate seasonally [1]. During the migration journey, the birds cross mountains, seas and rivers throughout different countries around the world, making them a potential transboundary vector for many pathogens [2]. Collectively, migratory birds usually have eight major migratory pathways around the world: Pacific Americas; Central Americas; Atlantic Americas; East Atlantic; Black Sea‐Mediterranean, East Asia-East Africa; Central Asia and East Asia-Australian [3]. However, East Atlantic; Black Sea‐Mediterranean, East Asia-East Africa flyways constitute the Palearctic-African flyway to be the largest flyway throughout the world. Egypt has a unique geographical location to be the heart of the Palearctic African flyway, that is why every year, millions of birds visit Egypt during their normal migratory flyway [4,5].

Migratory quails and ducks may be reservoirs for a wide array of zoonotic pathogens, including bacterial, viral, mycotic and parasitic ones, they act as either healthy carrier or host for such pathogens [6]. Migratory quail is also called common quail (*Coturnix coturnix*) belonging to migratory Galliformes; it is distributed along Eurasia during their breeding season; it can also be a reservoir for many enteric pathogens like *Campylobacter* spp. and Shiga toxin-producing *Escherichia coli* [7].

There are many species of migratory ducks coming to Egypt from Europe, such as *Anas acuta* (Northern Pintail) which is found to be a reservoir for the avian influenza virus [8], *Anas clypeata, Anas crecca, Anas platyrhynchos and Fulica atra* [9]. *Anas crecca* is considered an abundant duck species in Europe; they have many migration routes. One of them is Nile river south to East Africa pathway that occurs in the autumn. Whilst from December to March most of *Anas crecca* (common teal) ducks migrate to south and West Europe, few numbers of them were reported in North Africa and cross the Sahara desert [10,11].

*Escherichia coli* is a normal commensal in the gut of birds and animals; some migratory birds may carry pathogenic strains of *E. coli* likewise, enteropathogenic *E. coli* and Shiga toxin-producing *E. coli* [12]. In Italy, STEC was isolated from wild ducks and live common quail faecal matter, which indicates the potential role of migratory ducks and quails in the transmission of STEC [7,13]

Shiga toxin-producing *E. coli* (STEC), which is also known as verotoxigenic *E. coli*, was first identified by Konowalchuk [14]. However, it is introduced to human medicine in 1982 after human cases of haemorrhagic colitis caused by *E. coli* O 157: H7 [15,16]. STEC strains produce two types of Shiga toxins that are antigenically different; Shiga toxin type 1 (Stx1) and Shiga toxin type 2 (Stx2). Stx2 is known to be more potent than Stx1 and may cause fatal human illnesses such as haemolytic uraemic syndrome [17,18]. Therefore, STEC is considered as a foodborne pathogen with severe clinical outcomes among humans [19]. Migratory birds during their migration journey can act as carriers of antimicrobial resistant and multidrug resistant pathogenic bacteria like *E. coli* [20,21].

There was scarce data about the occurrence of STEC among migratory birds during their flying pathway.

Accordingly, the aims of the current study are as follows: (1) to investigate the occurrence of STEC among the examined migratory ducks and quails that are visiting Egypt during their migratory pathway, (2) evaluation of antimicrobial resistance of the obtained STEC isolates, (3) sequencing and BLAST analysis of the obtained genes (*stx1* and/or *stx2*), (4) phylogenetic analysis to highlight the public health importance of such strains.

## 2. Materials and methods

### 2.1. Ethical statement

The study protocol was approved by the Institutional Animal Care and Use Committee (IACUC), faculty of veterinary medicine, Cairo University. Approval number: Vet Cu 28/04/2021/298.

### 2.2. Samples

Cloacal swabs were collected from 349 migratory birds directly after catching them at Damietta governorate, Egypt during the migratory season (from September 2018 to January 2019). The birds were captured alive by expert hunters using the nests. The species of birds are shown in Table 1. The collected cloacal swabs were inserted in sterile tubes containing Cary-Blair transport medium (Liofilchem s.r.l, Italy) and transported to the laboratory in icebox with minimum delay. The distribution of samples among different examined migratory birds is shown in Table 1.

**Table 1.** Number of samples from the examined different species of migratory birds

Bird species	English name	No. of samples
*Anas clypeata*	Northern shoveler	8
*Anas acuta*	Pintail	70
*Anas crecca*	Common Teal	109
*Anas platyrhynchos*	Mallard	6
*Fulica atra*	Common Coot	16
*Coturnix coturnix*	Quail	140

The identification of the birds has been done according to Carboneras [42].

### 2.3. Isolation and identification of STEC

Swabs were streaked onto STEC CHROMagar™ medium (Paris, France), and incubated for 24 hours at 37°C. The suspected colonies were sub cultured for obtaining pure culture [22]. Afterwards, Gram’s staining and conventional biochemical tests were conducted according to Kavitha and Devasena [23] and using RapID ONE System for identification of *Enterobacteriaceae* (Oxoid, UK).

### 2.4. Molecular identification of STEC

PCR reaction was performed to detect the *stx1, stx2* and *eae* genes.

#### 2.4.1. DNA extraction

DNA was extracted from suspected STEC colonies enriched in brain heart infusion broth using G-spin Total DNA extraction kit (iNtRON, Korea) and the procedures were done according to the manufacturer’s guidelines; then, the extracted DNA was stored at −20°C for further investigation.

#### 2.4.2. The PCR assay

Uniplex PCR was carried out, targeting *stx1, stx2* and *eae* genes using specific primer sets. *Stx1* gene primers: F (5′-ACACTGGATGATCTCAGTGG-3′) and R (5′-CTGAATCCCCCTCCATTATG-3′) amplify 614bp whereas *stx2* gene primers: F (5′-CCATGACAACGGACAGCAGTT-3′) and R (5′-CCTGTCAACTGAGCAGCACTTTG-3′) target 779bp according to Dipineto *et al*. [24]. While *eae* gene primers: F (5′-CTGAACGGCGATTACGCGAA-3′) and R (5′-CCAGACGATACGATCCAG −3′) target 917bp according to Reid *et al*. [25], and all primers were synthesized by “Willowfort, UK”. The PCR reaction mixture for *stx1, stx2* and *eae* genes comprised 12.5 ul Emerald Amp GT PCR master mix (2x premix), 5.5 μl PCR grade water, 1 μL forward primer (20 pmol), 1 μL reverse primer (20 pmol) and 5 μl DNA with a total reaction volume 25 ul for each reaction.

The amplification conditions for both *stx1*and *stx2* genes were carried out according to Dipineto *et al*. [24] with the following thermal profile: initial denaturation at 94°C for 5 min, then 35 cycles of; denaturation 94°C for 30 sec, annealing at 58°C for 40 sec, extension at 72°C for 45 sec followed by a final extension at 72°C for 10 min. However, the cycling condition of the *eae* gene was conducted according to Reid et al. [25]: initial denaturation at 95°C for 5 min followed by 35 cycles of; denaturation at 95°C for 40 sec, annealing at 58°C for 1 min, extension at 72°C for 1 min then final extension at 72°C for 5 min.

A T3 Thermal cycler (Biometra, Germany) PCR system was used for PCR reactions; gel electrophoresis step was done for PCR products (10 uL) in 1.5% agarose with DNA ladder (100 bp) using a gel documentation system (Alpha Innotech, USA).

### 2.5. DNA sequencing

The PCR product was purified using QIAquick PCR Product extraction kit (Qiagen Inc. Valencia, CA) according to manufacturer’s instructions, then amplicons from purified PCR products of *stx1* and *stx2* genes were sequenced using Applied Biosystems 3130 automated DNA Sequencer (ABI, 3130, USA).

### 2.6. Sequence identity and phylogenetic analysis

**Figure 1.**
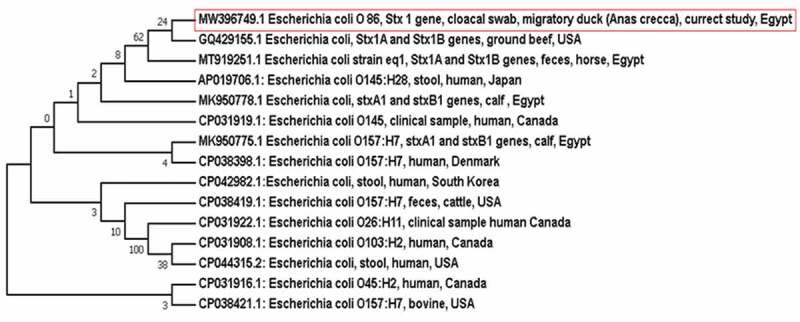
Neighbour-joining phylogenetic bootstrap consensus tree shows the evolutionary history and the genetic relatedness of the obtained *stx1*gene sequence of STEC recovered from a migratory duck and other sequences retrieved from the GenBank records

**Figure 2.**
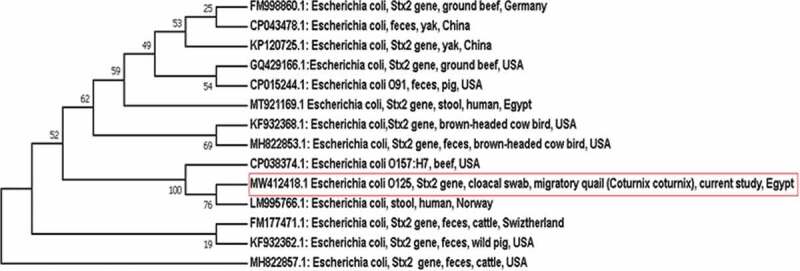
Neighbour-joining phylogenetic bootstrap consensus tree clarifies the evolutionary history and the genetic relatedness of the obtained *stx2* gene sequence of STEC recovered from a migratory quail and other sequences retrieved from the GenBank records

Both *stx1* and *stx2* gene sequences were analysed using nucleotide BLAST on the NCBI website (www.ncbi.nlm.nih.gov/BLAST) to identify the most similar sequences available in the GenBank. Afterwards, each *stx1* and *stx2* gene sequence was aligned against some selected similar sequences (from human cases as well as animal hosts) retrieved from the GenBank. The alignment of the sequences was done using Clustral W Multiple Alignment (BioEdit 7.0.9) and two phylogenetic trees have been constructed using the neighbour-joining method based on *stx1* and *stx2* partial gene sequences, respectively, with MEGA 7 software (version 7.0.26). The phylogenetic clusters were conducted using bootstrapping analysis with 500 replicates as shown in Figures (1 and 2).

### 2.7. Serological identification of STEC isolates

The STEC isolates were serotyped according to Ewing *et al*. [26] using slide agglutination technique, it was done in the reference laboratory, Dokki, Egypt.

### 2.8. Antibiotic susceptibility testing of STEC isolates

Antibiotic susceptibility testing of STEC isolates using 17 antibiotic disks from different classes was done using the disk diffusion method. First, bacterial suspensions were prepared from pure culture using 0.5 McFarland standard, then plates of Mueller–Hinton agar medium were prepared, and then bacterial suspensions were swabbed on the Mueller–Hinton plate. Finally, the antibiotic disks were placed on the inoculated plates according to Kirby–Bauer disk diffusion susceptibility test protocol and the Clinical and Laboratory Standards Institute (CLSI) guidelines [27,28], antimicrobial agents used in this study with referring to their antimicrobial classes were as follows: penicillins (ampicillin 10 ug), aminoglycosides (amikacin 30 µg and gentamicin 10 µg), phenicols (chloramphenicol 30 µg), quinolones (ciprofloxacin 5 µg), carbapenems (meropenem 10 µg, ertapenem 10 ug, and imipenem 10 ug), tetracyclines (tetracycline 30 µg, doxycycline 30 ug), cephalosporins (ceftriaxone 30 µg, cefpodoxime10 ug, ceftazidime 30 ug, cefepime 30 ug, and cefotaxime 30 ug), macrolides (azithromycin 15 ug), and nitrofuran (nitrofurantoin 300 ug).

### 2.9. Nucleotide sequence accession numbers

The obtained *stx1* and *stx2* gene sequences were deposited in the GenBank under the accession numbers: MW396749.1 and MW412418.1 for *stx1* gene and *stx2* gene sequence, respectively.

## 3. Results

Out of the 349 examined migratory birds, two yielded STEC (1 strain from duck was serotyped as O86 and the other from quail was serotyped as O125) giving an overall occurrence 0.57%. The occurrence of STEC among the migratory ducks was 0.48% with an occurrence rate 0.92% among *Anas crecca* ducks, whereas that among quails was 0.71%. The duck strain carried *stx1* gene, while the quail strain possessed *stx2* gene, but none of the STEC isolates was positive for *eae* gene (Table 2). The antibiotic resistance patterns of both STEC isolates are displayed in (Table 3). STEC O86 was resistant to ampicillin, ceftazidime, ceftriaxone, cefepime, meropenem and chloramphenicol, whereas STEC O125 was resistant to ampicillin, ceftriaxone, meropenem, azithromycin and chloramphenicol. Moreover, the identity of the obtained DNA sequences of *stx1* and *stx2* genes based on BLAST analysis is demonstrated in (Table 4). Furthermore, the phylogenetic bootstrap consensus trees of *stx1* and *stx2* genes showed the genetic relatedness of the sequences from the obtained isolates with others from both human and animal hosts.

**Table 2.** Occurrence of STEC and related genes among the examined migratory birds

Bird	Species	Tested birds	Positive birds	Percentage %	*Stx1* gene	*Stx2* gene	*Eae* gene
Quails	*Coturnix coturnix*	140	1	0.71	-ve	+ve	-ve
Migratory ducks	*Anas crecca*	109	1	0.92	+ve	-ve	-ve
	Other species	100	0	0	-ve	-ve	-ve
	Total of the examined ducks	209	1	0.48	+ve	-ve	-ve
Total		349	2	0.57

**Table 3.** The antibiotic resistance patterns of both STEC isolates

STEC		Penicillins		Cephalosporins					Carbapenems			Aminoglycosides		Macrolides	Tetracycline		Quinolones	Phenicols	Nitofurans
	amp		cpd		caz	cro	cpm	ctx	mrp	etp	ipm	gen	ak	azm	te	do	cip	c	nit
O86	R		R		R	R	R	S	R	S	S	S	S	S	S	S	S	R	S
O125	R		R		S	R	S	S	R	S	S	S	S	R	S	S	S	R	S

S = susceptible, I = intermediate, R = resistant.

amp (ampicillin 10ug), gen (gentamicin 10 ug), cip(ciprofloxacin 5 µg), cpd (cefpodoxime 10 µg), caz (ceftazidime 30 µg), cro (ceftriaxone 30 µg), cpm(cefepime 30 µg), ctx (cefotaxime µg), mrp (meropenem 10 µg), etp (ertapenem 10 µg), IPM (imipenem 10 µg), ak (amikacin 30 µg), azm (azithromycin 15 µg), te (tetracyclin 30 µg), do (doxycycline 30 µg), c (chlo-ramphenicol 30 µg), nit (nitrofurantoin 300 µg).

**Table 4.** The identity of the *stx1* and *stx2* gene sequences after BLAST analysis

The obtained gene sequence	*E. coli* serotype	Migratory bird species	Identity %	Accession number	Country	Source
*Stx1* MW396749.1	O86	Duck (*Anas crecca*)	100	CP027586.1	USA	Human stool
			100	GQ429155.1	USA	Ground beef
			100	LM996514.1	Norway	Human stool
*Stx2* MW412418.1	O125	Quail (*Coturnix coturnix)*	100	MT921169.1	Egypt	Human stool
			99.72	CP038374.1	USA	beef
			99.72	MH822853.1	USA	Faeces of brown-headed cow bird

## 4. Discussion

Migratory birds may transmit many emerging pathogens, including a diversity of zoonotic ones likewise avian influenza, *Salmonella* and *E. coli* with a great public health importance [29]. The results of the current study revealed that the overall occurrence of STEC among the examined migratory birds was 0.57%. The occurrence among the examined ducks was 0.48%, while STEC was detected only among *Anas crecca* duck species with an occurrence rate 0.92%, with such result was lower than that obtained by Fadel *et al*. in Egypt, who isolated STEC with occurrence rate 10% (1/10) among the examined *Anas crecca* [30] and Bertelloni *et al*. in Italy, who recovered STEC with occurrence rate 4.5% (1/22) [13]. The higher results obtained by such others may be owed to a different environment or using different diagnostic methods. The isolated STEC strain from a migratory duck in our study possessed *stx1* gene, which agreed with Bertelloni *et al*. [13] while Fadel *et al*. [30] found both *stx1* and *stx2* genes in only one positive duck sample. On the other hand, the occurrence of STEC among the examined migratory quails was 0.71%, which is considered lower than that found by Dipineto *et al*. 5.7% [7]. Serotyping of the obtained strains revealed that the duck strain was *E. coli* O86, while the quail one was *E. coli* O125. Interestingly, *E. coli* O125 was thought to be enteropathogenic *E. coli*; however, some recent studies underline the occurrence of *stx* genes in such serotype [31–33]. Also, strains of *E. coli* O86 usually belong to the enteropathogenic *E. coli* group, although a recent study classified strains of *E. coli* O86 as Shiga toxin-producing enteroaggregative *E. coli* which has a great public health implication [34,35].

STEC has been incriminated in many outbreaks among humans throughout the world. According to the World Health Organization (WHO) report in 2010 [15], there are more than 1 million cases and 100 deaths occurred due to STEC infections. Although STEC O157: H7 is the most important serotype, several non-O157 STEC serotypes have emerged in the last few years to be a cause of serious human illnesses [15].

The public health significance of the occurrence of STEC among the migratory birds arises from the potential for direct transmission of these pathogens to humans through contact with birds after hunting. Also, ducks often shed STEC in their droppings, contaminating the lakes and water streams where they reside during the migration journey, and thereby contaminated water may be a source of infection to humans and domestic animals [21]. In this regard, it has been found that migratory birds may share in the dissemination of *E. coli* in the environment and transmit such strains to dairy animals causing mastitis [36].

Seriously, both STEC isolates obtained in the current study are multidrug-resistant strains (MDR), as they showed resistance to antibiotics from four or five different classes [37].

Unexpectedly, both strains were resistant to meropenem that belong to the carbapenem antibiotic class, and according to the Centers for Disease Control and Prevention (CDC), *E. coli* strains that show resistance to one of the carbapenem antibiotics were considered as carbapenem-resistant *Enterobacteriacaea* (CRE) to be regarded as an urgent public health threat [38].

Noteworthy, the results of the BLAST analysis of the obtained sequences revealed that *stx1* gene sequence of STEC O86 isolated from the migratory duck showed 100% identity with those of STEC recovered from humans in the USA and Norway as well as ground beef from the USA. On the other hand, *stx2* gene sequence of STEC O125 isolated from migratory quails demonstrated 100% identity with that of STEC strain isolated from human in Egypt and 99.72% identity with those of STEC recovered from brown-headed cowbird and ground beef from the USA. Furthermore, the results of the phylogenetic analysis came to confirm those of BLAST analysis to highlight the high genetic relatedness of the obtained sequences of STEC isolates from the migratory birds and those circulated in the USA and North Europe. Phylogenetic analysis showed that *stx1* gene of STEC isolated from *Anas crecca* duck was grouped in the same clade with *stx1* gene of STEC isolated from ground beef in the USA. However, *Anas crecca* ducks reside in North Europe, such similarity with ground beef in the USA may be owed to the fact that *Anas crecca* ducks may go abmigration during their migration journey to reach the USA [39,40]. Strikingly, *stx2* gene sequence of STEC isolated from quail was placed in the same clade with *stx2* gene sequence of STEC isolated from human in Norway, which is the native breeding home of quail (*Coturnix coturnix*), according to birdlife distribution map of quails [41], to point out the ability of such birds to transmit STEC originated from their native breeding home country to other countries throughout their migration journey.

## 5. Conclusion

This study sheds more light on the possible role that may be played by migratory birds in the transmission of exotic multidrug-resistant STEC strains with great public health concern during their migratory pathway. Hence, migratory ducks and quails may be considered as an overseas transmitter for such dangerous pathogens. Further studies are required in the same vein to provide more knowledge about the occurrence and the characteristics of different pathogens that may be carried by the migratory birds. In the meantime, public health awareness should be implemented for people at risk in order to take all safety precautions during handling of migratory birds.
